# Point-of-Care Diagnostics for Farm Animal Diseases: From Biosensors to Integrated Lab-on-Chip Devices

**DOI:** 10.3390/bios12070455

**Published:** 2022-06-26

**Authors:** Georgios Manessis, Athanasios I. Gelasakis, Ioannis Bossis

**Affiliations:** 1 Laboratory of Anatomy and Physiology of Farm Animals, Department of Animal Science, Agricultural University of Athens (AUA), Iera Odos 75 Str., 11855 Athens, Greece; gmanesis@aua.gr (G.M.); gelasakis@aua.gr (A.I.G.); 2 Laboratory of Animal Husbandry, Department of Animal Production, School of Agriculture, Faculty of Agriculture, Forestry and Natural Environment, Aristotle University of Thessaloniki, 54124 Thessaloniki, Greece

**Keywords:** point-of-care diagnostics, lateral flow assays, lab-on-chip devices, micro total analysis systems, microfluidics, farm animal diseases, biosensors, legislation and regulation, challenges of point-of-care testing, future perspectives

## Abstract

Zoonoses and animal diseases threaten human health and livestock biosecurity and productivity. Currently, laboratory confirmation of animal disease outbreaks requires centralized laboratories and trained personnel; it is expensive and time-consuming, and it often does not coincide with the onset or progress of diseases. Point-of-care (POC) diagnostics are rapid, simple, and cost-effective devices and tests, that can be directly applied on field for the detection of animal pathogens. The development of POC diagnostics for use in human medicine has displayed remarkable progress. Nevertheless, animal POC testing has not yet unfolded its full potential. POC devices and tests for animal diseases face many challenges, such as insufficient validation, simplicity, and portability. Emerging technologies and advanced materials are expected to overcome some of these challenges and could popularize animal POC testing. This review aims to: (i) present the main concepts and formats of POC devices and tests, such as lateral flow assays and lab-on-chip devices; (ii) summarize the mode of operation and recent advances in biosensor and POC devices for the detection of farm animal diseases; (iii) present some of the regulatory aspects of POC commercialization in the EU, USA, and Japan; and (iv) summarize the challenges and future perspectives of animal POC testing.

## 1. Introduction

The human demand for animal-based food products is expected to double by 2050 [1]. At the same time, the imbalance between food demand and supply is predicted to widen even further, necessitating adjustments in human dietary habits to conserve natural resources [2]. Thus, maintaining a sustainable and sufficient supply of animal protein to meet the requirements of a balanced diet will be a challenging task [3].

Animal diseases affect the sustainable production of animal-derived foods by decreasing the, potential output and cost effectiveness and by deteriorating overall animal health and welfare. Industrialized and high-input farming systems ramp up productivity, yet they are more susceptible to disease outbreaks due to their higher stocking density [4]. Trade globalization and the scarcity of surveillance systems for animal diseases further contribute to the spread of diseases [5,6]. Consequently, the effective control of diseases is important to minimize their socioeconomic impact and safeguard animal-derived food supply.

The early and reliable diagnosis of animal diseases is critical for the implementation of evidence-based countermeasures and the minimization of disease spread. The diagnosis of animal diseases often requires laboratory confirmation, which is based on various PCR protocols, immunoassays and/or cell cultures, making well-equipped laboratories and trained personnel indispensable [7]. This process results in long turnaround times (from sampling to results) that can vary from days to weeks, often making diagnosis non-concurrent with the progress of the disease [8]. Additionally, laboratory confirmation is costly and unsuitable for resource-limited settings.

To cover this gap in animal disease diagnostics, the use of point-of-care (POC) devices and tests has been proposed. POC diagnostics are analytical devices and other tests that provide rapid diagnostic capabilities, without the need for core laboratory facilities [9]. The World Health Organization (WHO) has set the criteria for an ideal POC application under the acronym ASSURED, which stands for: (1) affordable, (2) sensitive (minimum number of false negatives), (3) specific (minimum number of false positives), (4) user-friendly (simple to perform), (5) rapid and robust, (6) Equipment-free, and (7) deliverable to those who need them [10].

The first POC diagnostics were developed for human medicine and targeted various biomarkers and infectious diseases. Special emphasis was placed on POC tests for the diagnosis of infectious diseases, such as malaria and HIV, which plague developing regions. Following in these footsteps, various biosensors and POC applications for economically significant diseases and zoonoses started to emerge. The COVID-19 pandemic has popularized POC testing on a global scale [11]. However, a similar trend has not yet been observed in POC diagnostics for farm animal diseases. For example, only two POC devices from a total of 14 diagnostic kits for 11 animal diseases that have been registered to OIE (https://www.woah.org/en/what-we-offer/veterinary-products/diagnostic-kits/the-register-of-diagnostic-kits/ accessed on 17 June 2022). The most recent POC devices target a small number of animal pathogens that are either economically important (African swine fever, classical swine fever, porcine reproductive and respiratory syndrome, rinderpest, foot and mouth disease, and bluetongue disease) or have zoonotic potential (avian and swine influenza, *Salmonella* spp., *Brucella* spp., *Escherichia coli*, and *Campylobacter* spp.).

The objectives of this review are: (i) to summarize the foundations of POC diagnostics and the recent achievements in biosensor development; (ii) to present the available biosensors and POC diagnostics for farm animal diseases; and (iii) to discuss the regulative framework, challenges, limitations, and future directions of POC testing in animal production.

## 2. Categories of POC Devices

### 2.1. Paper-Based POC Devices

The fabrication of paper-based diagnostic tests relies on two basic materials, cellulose and nitrocellulose [12]. Cellulose is a linear macromolecule composed of glucose units; it is fibrous, hydrophilic, biodegradable, and insoluble in water and most organic solvents. Nitrocellulose is the product of cellulose nitration, a process that strengthens porosity and makes cellulose hydrophobic [13]. Porosity, surface chemistry, and optical properties are the most important characteristics of paper-based diagnostic devices. Surface chemistry affects the immobilization and absorption of molecules and, along with porosity, determines the fluidic properties of paper. Optical properties affect the colorimetric and fluorescent readouts. Polymeric additives are commonly used to improve these properties of cellulose and nitrocellulose paper [14].

The detection of analytes is usually mediated via chemical, electrochemical, electrochemiluminescence, and chemiluminescence mechanisms and image analysis. The chemical detection mechanism exploits precipitation, acid–alkali (pH indicators) and redox reactions, and molecular and enzymatic dyes to generate colour changes. Nanoparticles (gold, silver, latex, carbon dots, etc.) can be labeled with antibodies, antigens, aptamers, or oligonucleotides, enabling the visual observation of biorecognition events. In enzyme-mediated colorimetric detection, redox indicators, combined with oxidases, peroxidases, and phosphatases are utilized. Redox indicators produce coloration, a process influenced by the contrast between the reduced/oxidized forms of the substrate and the background color of the paper material [15]. Quantification with color-coded charts is possible, but it involves challenges, such as the uneven distribution of colour, the linearity of the response, and subjective color assessment. The electrochemical detection mechanism (e.g., in glucose meters) is based on both redox reactions (electron transfer between, for example, enzymes and nanoparticles) and non-redox reactions that alter the electrical properties of the sample (impedance, resistance, conductance, and potential). The reactions can be transduced by low-cost electrodes, allowing quantification and enabling high sensitivity and selectivity [14]. Electrochemical detection is fast, sensitive, and independent of ambient light and color deterioration due to the properties of paper. However, it requires reading equipment, increasing the cost and complexity of the devices it involves. The electrochemiluminescence method is based on the light emissions of electronically excited intermediates to enable readouts. These intermediates are electrochemically generated via exergonic reactions. Cameras featuring photomultipliers can be used for signal amplification, detection, and quantification. Typically, cameras must operate in dark conditions to avoid ambient-light interference [16]. Chemiluminescence is based on chemically generated luminescence. Hydrogen peroxide and either luminol or rhodamine are commonly used in chemiluminescence to produce optical signals. The method is suitable for the sensing of various biological analytes (cells, bacteria, and DNA/RNA) and is compatible with microfabrication [17]. Finally, image analysis, performed by low-cost smartphones, can be combined with the above detection mechanisms to provide better signal measurements [18].

However, these methods do not always suffice for robust measurements. Signal enhancement and increases in colour intensity via enzymatic reactions or the accumulation of nanoparticles (gold, silver, latex particles, quantum dots, up-converting phosphor reporters (UCP), carbon nanotubes and particles, platinum nanoparticles, lanthanide, SiO_2_ nanoparticles, super-paramagnetic nanoparticles, etc.) is common in paper-based POC devices to improve their performance [15,19]. Paper inhibits electrical signals. Thus, the doping of paper with conductive polymers, nanocomposites, and graphene, aiming to transforming it into a semiconductor or a conductor, is crucial for signal amplification in electrochemical detection [20,21].

#### 2.1.1. Dipstick and Strip Tests

The basic formats of paper-based POC devices are dipstick and strip tests and lateral flow assays (LFAs). Dipstick and strip tests are based on colorimetric measurements, allowing the semi-quantitative determination of the analytes using colour-coded charts. Strip and dipstick tests are commonly used for the detection of various analytes or the assessment of physicochemical properties in biological fluids, such as in milk and urine analyses. For example, dipstick tests have been developed for the detection of antibiotics in animal-derived food products and ketone bodies for the monitoring of diabetic pets or the diagnosis of ketosis in cattle [22,23,24]. These tests are rapid, convenient, and cheap, and they can be carried out by untrained personnel, on-site. However, in many cases, the confirmation of the diagnosis using other assays or devices may be necessary [24].

#### 2.1.2. Lateral Flow Assays (LFAs)

**Figure 1:**
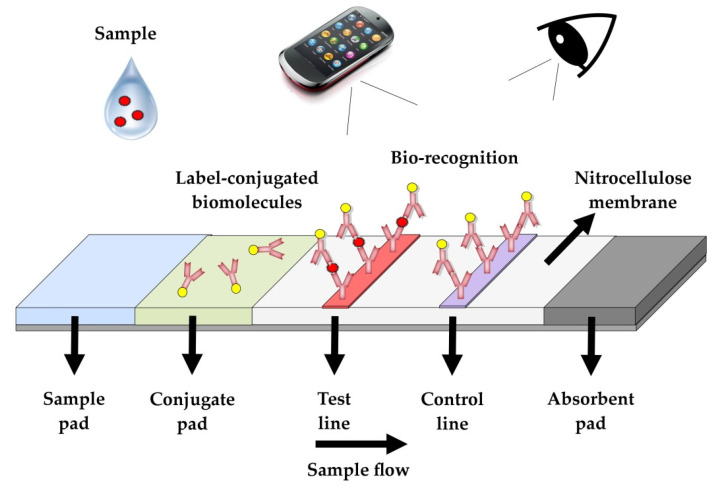
Principle of LFA sandwich format.

LFAs are qualitative or semi-quantitative diagnostic tests consisting of a sample application pad, a conjugate pad, a membrane for detection (commonly nitrocellulose), and an absorbent pad (Figure 1). The adjacent components of the test are overlapped for the coordination of the liquid flow [25]. The pads are usually made from different materials, such as nitrocellulose, glass-fiber paper, and fused silica. A protective plastic cage is used to limit contamination, the evaporation of reagents and samples, and susceptibility to light [16]. LFAs operate in a simple way. The sample pad is pretreated with a buffer solution, which is mixed with the sample to improve the performance of the reactants during the assay [26]. As the mixture flows, it carries conjugated (labeled) particles, which are preloaded on the conjugate pad. The conjugated particles are captured on the control and test zones of the membrane, enabling the detection of the analyte [25]. The dispersion of liquid samples is based on the capillary forces generated by the absorbent pad.

Two main formats of LFA tests are used: the sandwich and the competitive formats. In the sandwich format, the analytes react with the conjugated particles to form a complex, which is then captured by immobilized molecular recognition elements (MREs, e.g., antibodies) on the test line via the remaining binding sites of the analyte. Free conjugated particles can flow further, reacting with other MREs to form the control line [19]. In the competitive format, the conjugated particles can react with MREs on both the control and test lines. On the test line, the analyte and the conjugated particles compete for the binding sites of the capturing molecules. As a result, conjugated particles do not aggregate on the test line. Sandwich formats are preferred when the analyte has multiple binding sites (e.g., viruses, bacteria, etc.), while competitive formats are used for the detection of analytes with a single binding site [19].

LFAs are simple and rapid and have been popularized due to the COVID-19 pandemic [11]. Relevant tests for farm animals are used, mainly for zoonotic diseases with high economic impact, such as foot and mouth disease (FMD) and rinderpest, which require rapid diagnosis and immediate intervention [27,28]. LFAs have been successfully applied in the food industry for the detection of food-borne pathogens and other unwanted and dangerous substances (e.g., mycotoxins) in animal feed or animal products. The limitations of LFA testing include inferior performance compared with laboratory-based assays, the increased likelihood of misuse when handled by unskilled personnel, and qualitative or semi-quantitative measurements. In recent years, advancements in instrumentation have improved their quantitative capabilities to some extent [29].

### 2.2. Microfluidic POC Devices

Microfluidic devices utilize a network of microchannels for the analysis of fluids at the microliter or nanoliter scale. The analytical procedure usually takes place within microfluidic chambers or specially formed microfluidic channels. The laminar flow of fluids is achieved either passively (capillary forces) or actively with pumping mechanisms in fully integrated devices [30]. During the last 30 years, microfluidics have greatly evolved, mainly due to advances in microfabrication technologies [31].

Microfluidic devices can be portable and require small quantities of sample and reagents. Therefore, they have the potential to be easier to use and more cost-effective than laboratory testing. The all-in-one approach of integrated microfluidic devices makes them more attractive in comparison with traditional laboratory techniques due to their rapidity, the reduction in specialized labor that they entail, and the decreased risk of human error involved. These technologies are currently applied in blood biochemical analysis, in pathogen identification, and in the detection of environmental contaminants [29]. The main types of microfluidic device are micro total analysis systems (μTAS), also known as “lab-on-a-chip” (LOC) devices, and microfluidic paper-based analytical devices (μPAD).

#### 2.2.1. Micro Total Analysis Systems (μTAS)—Lab-on-Chip (LOC) Devices

**Figure 2:**
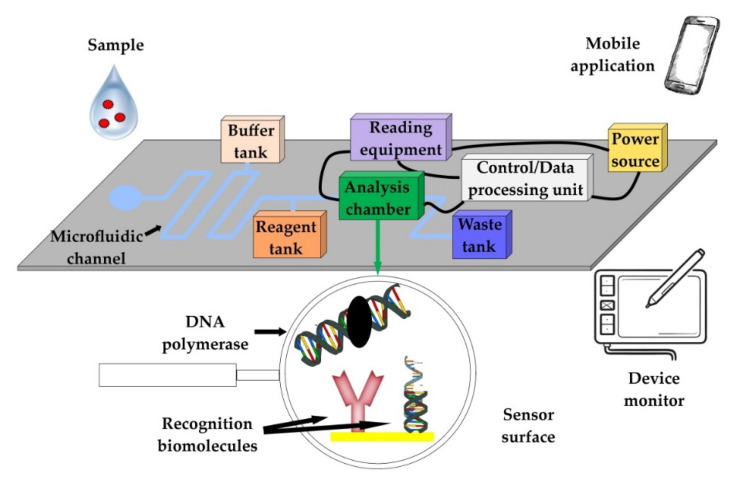
Concept and main components of fully integrated LOC devices. The detection chip (gray parallelogram and analysis chamber) is magnified for demonstration purposes.

LOC devices can perform different assays on a micro-scale (e.g., PCR, LAMP, RCA, etc.), since they can integrate all the analytical steps in a single platform [32]. For the development of these platforms, technological advances from the microelectronics industry have been exploited [33]. The basic core of the detection chips, where the biorecognition/analysis takes place, is constructed from glass, quartz, silicon, or polymeric materials, with the latter considered by many to have the most suitable mechanical, chemical, and thermal properties. Some of the most commonly used polymeric materials are polytetrafluoroethylene (PTFE), photosensitive silicon, poly(methylmethacrylate) (PMMA), and biopolymers, such as cellulose acetate [32,34]. The efficiency of detection chips depends on the microfluidic channel design, the overall construction of the apparatus, and the affinity and avidity of the main recognition elements on the chip. The later characteristics also determine, in most cases, the diagnostic performance of the devices. Their manufacturing is usually performed using soft lithography and 3D printing and requires the use of additional equipment for data extraction, signal processing, and monitoring [35]. The common methods of signal detection in LOC systems include light detection, magneto-resistive sensors (GMR), electrochemical detection, acoustic sound-wave detection, mass spectroscopy (MS), and nuclear magnetic resonance (NMR) [36]. The detection chips and any additional equipment are generally integrated into single devices (Figure 2).

#### 2.2.2. Microfluidic Paper-Based Analytical Devices (μPAD)

The idea behind the creation of μPADs originated from the Whiteside group at Harvard University, as an aftermath of the research performed on paper strips for pH determination [37]. These μPADs lie at the crossroads of paper-based and microfluidic devices and maintain the benefits of microfluidics, utilizing low-cost materials (paper) and simple production processes [38]. More specifically, μPADs facilitate various forms of microfluidic handling, such as transportation, sorting, mixing or separation in paper-based detection systems. This allows the integration of more complex assays than those found in LFAs and strip tests [39]. In addition, μPADs do not require an energy supply or other mechanical valves and pumps, as the fluids are mobilized by capillary forces [37]. These devices can be manufactured in two or three dimensions (2D or 3D) to transport fluids both vertically and horizontally [37]. In contrast to LFAs and strip tests, the formation of the microfluidic channels in μPADs requires the creation of hydrophobic barriers (the blocking of the paper pores). The hydrophobic patterning determines the length and width of the microfluidic channels, while the paper thickness determines their height [38]. Several methods of hydrophobic patterning are used. Wax printing and dipping, movable-type wax printing and wax screen printing offer low-cost, non-toxic, and disposable microfluidic devices; however, the resolution of the microfluidic channels is low and the cost of the required equipment for production is relatively high [40]. Wax printers deposit solid wax for patterning, whereas a heat source melts the wax to facilitate absorption by the paper sheets [41]. Another patterning method, inkjet printing, utilizes a single piece of equipment (printer) to spray hydrophobic materials (e.g., SU-8, PDMS etc.) on paper. It is a low-cost and rapid technique, suitable for commercial exploitation [42]. Inkjet etching refers to the spraying of solvent ink on solid polymeric material-covered paper. The dissolution of the polymeric material by the solvent ink reveals a hydrophilic microfluidic pattern due to the exposure of the paper. Flexograpfic printing is based on the application of polydimethyl-siloxane (PDMS) ink on paper. However, rapid, modified commercial press-printing equipment and a multi-step procedure are necessary for its implementation [40]. The procedure includes the fabrication of a photopolymerized (UV light) flexographic printing mold (FMold) and the transfer of the microfluidic pattern to an epoxy resin mold (ERMold). The deposition of PDMS on paper is facilitated by the ERMold [43]. Photolithography exploits the properties of low-cost, light-sensitive photoresistants. Non-polymeric solid substrates (e.g., glass, silicon, etc.) are covered with a photoresistant and then exposed to UV light through a high-resolution mask (plastic or glass film) for the creation of microfluidic channels. Based on the desired output, photolithography can vary from a rapid and user-friendly method requiring simple equipment, such as UV light and a heating plate, to a more complicated method, requiring trained staff, expensive, sophisticated equipment, and specialized infrastructure (clean rooms) [42]. Laser cutting utilizes a CO_2_ laser for the patterning of chromatography or nitrocellulose paper, coated with hydrophobic materials. The CO_2_ laser produces an infrared light beam for surface etching. This method is simple, but requires specialized equipment (CO_2_ laser, 2D graphics, etc.) [13]. Finally, alkyl ketene dimer (AKD)-heptane can be applied to reverse the hydrophilic properties of paper. After the application of AKD, a heating step at 100 °C for 45 min is required to cure the AKD–heptane. The creation of hydrophilic patterning is achieved with plasma treatment; however, this method is costly [40].

**Figure 3:**
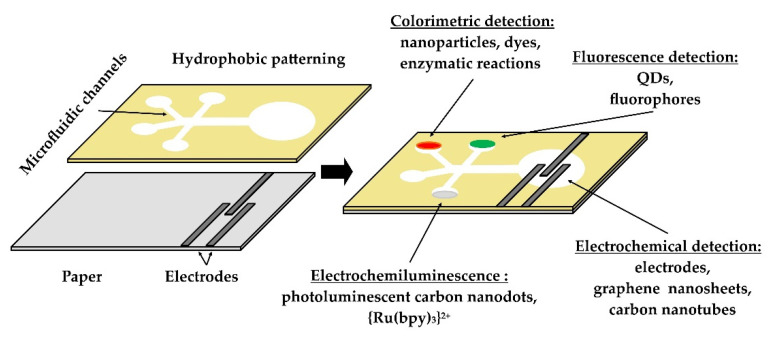
Concept and popular detection methods of paper-based microfluidic devices. The hydrophobic patterning determines the fluidic properties of these devices.

The detection mechanisms of μPADs include potentiometric, fluorimetric and colorimetric sensors, fluorescent quantum-dot nanoparticles and metal complexes, thermal (calorimetric) methods, electrochemiluminescence (ECL), and enzymatic methods (Figure 3) [44,45,46,47,48,49]. Paper-based electrochemical microfluidic devices (μPEDs) that utilize electrodes made from conducting inks (carbon or metal) through screen printing, inkjet printing, or pencil-drawing appear to be promising (Figure 3) [50]. These μPEDs are based on redox reactions and achieve a specificity similar to colorimetric reactions. Materials such as AuNP carbon nanotubes and graphene nanosheets are employed to modify the electrodes and increase sensitivity [14].

#### 2.2.3. Applications of Microfluidic Technologies

Microfluidic POC diagnostic devices can be divided into three major categories: nucleic-acid-based, protein-based, and cell-based applications. PCR is the most commonly used nucleic acid amplification technology; however, is labor-intensive and requires specialized equipment (thermocyclers). Therefore, research is focused on alternative isothermal amplification technologies, such as loop-mediated isothermal amplification (LAMP), nucleic-acid-sequence-based amplification (NASBA), helicase-dependent amplification (HDA), and recombinase polymerase amplification (RPA) for POC applications. Nucleic-acid detection techniques rely on fluorescence, colorimetry, or chemiluminescence. Amplification products can be detected visually or by smartphones. Isothermal amplification technologies integrated into LOC devices have been exploited for the detection of pathogens of veterinary importance, such as *Cryptosporidium parvum*, *Escherichia coli*, *Salmonella typhimurium*, and suid Herpesviruses [29].

Protein-based applications are relatively simpler and faster than nucleic-acid-based applications, as cell/viral lysis and nucleic acid purification are not required. Additionally, they require minimum sample pre-treatment and user involvement, thus facilitating the development of practical, commercially successful diagnostic devices [51]. A relevant POC application for farm animal diseases is a magnetic bead-based microfluidic device for the detection of antibodies against *Mycobacterium avium* subsp. *paraturbeculosis* and the diagnosis of Johne’s disease in cattle [52]. Cell-based microfluidic devices are mainly applied in human healthcare (e.g., whole-blood microfluidic cell counters and CD4 counters for HIV-infected patients) [51]. In the field of veterinary POC diagnostics, a lab-on-chip device has been developed for the detection of mastitis based on the measurement of neutrophil activity [53].

## 3. Biosensors in Animal Production

Biosensors are case-specific sensors that recognize analytes that are indicative of a given microorganism or condition. Analyte recognition is facilitated by immobilized sensing elements known as bioreceptors. Commonly used bioreceptors are monoclonal antibodies, RNA, DNA, glycans, lectins, enzymes, cofactors, tissues, and whole cells [52]. Biosensors can be divided to immunosensors, genosensors, non-enzymatic receptor sensors, enzymatic sensors, and whole-cell sensors. The interaction between the analyte and the bioreceptor is converted to a signal via a transductor [54]. The received signal is analyzed, allowing the qualitative and quantitative assessment of the analyte. An efficient biosensor needs to be capable of detecting the analyte regardless of the origin or the complexity of the biological sample to assure the robustness of bioassays [55]. In terms of signal transduction, biosensors can be classified to electrochemical, optical, piezoelectric, magnetic, thermal, radioactive, and mechanical sensors.

During the last 10 years, a staggering number of biosensors have been developed [56]. However, only a relatively small fraction of these biosensors target analytes that are relevant to animal production. In the following subsections, the available biosensors in animal production are classified based on signal-transduction methods.

### 3.1. Electrochemical Biosensors

**Figure 4:**
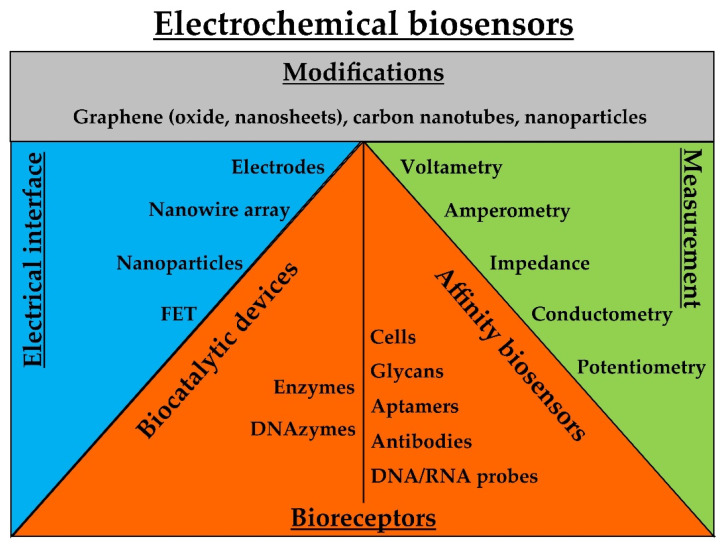
The basic components of electrochemical biosensors. The interaction of the targeted analyte with the bioreceptor causes an electrochemical change that can be transduced to measurable signals via the electrical interface. Nanomaterials and nanoparticles are used to improve the performance of the biosensors.

Electrochemical biosensors are capable of combining sensitive electroanalytical methods with the bioselectivity of certain molecules. Biorecognition-induced catalytic or binding events produce electrical signals that are monitored by transducers. The two major classes of electrochemical biosensors include biocatalytic devices and affinity sensors [57]. Amperometry and electrochemical impedance spectroscopy dominate the signal transduction techniques these devices. Recent developments in nanomaterials, such as graphene and carbon nanotubes, have popularized electrochemical detection [58]. Electrochemical technology is suitable for the production of low-cost, portable, and easy-to-use devices [58]. In this subsection, electrochemical biosensors are further classified to biocatalytic and affinity sensors (Figure 4).

Biocatalytic sensors combine the selective recognition and catalytic properties of enzymes with electrochemical transducers. Enzymes are immobilized on electrodes to modify their properties and catalyze the formation of electroactive products [59]. For instance, an electrochemical enzyme-based sensor utilizing superoxide dismutase has been developed for the detection of neutrophil excreted O_2_^· −^ radicals in mastitic milk [53]. Beyond classic enzymatic reactions, a peroxidase-mimicking DNAzyme coupled with rolling circle amplification (RCA) has been used for the detection of *Escherichia coli*. Electrodes functionalized with anti-*E. coli* antibodies were used to immobilize bacterial cells on a sensor’s surface. Probes containing anti-*E. coli* aptamers and primer sequences complementary to other secondary circular probes with two G-quadruplex units were used to both couple with immobilized bacterial cells and initiate RCA elongation through secondary probes. The coupling of the RCA with a DNAzyme leads to the formation of G-quadruplex oligomers on electrodes, which fold into G-quadruplex/hemin complexes in the presence of K^+^ and hemin. These complexes display strong catalytic activity toward H_2_O_2_, generating electrochemical signals. The biosensor had a detection limit of 8 CFU/mL and a detection range of five orders of magnitude [60]. Graphene and nanoparticles can be used to modify transducers and improve the performance of biocatalytic sensors. For example, a ruthenium bipyridyl complex has been coupled with graphene oxide nanosheets. The nanosheets were modified in sequence with lipoxygenase for the detection of non-esterified fatty acids (NEFA) in serum samples, achieving a sensitivity of 40.5 µA/mM and linear responses in a detection range of 0.1–1.0 mM [61].

Affinity sensors exploit electrodes, functionalized with various biomolecules, as the main transducing elements. In a previous study, aptamers immobilized on gold interdigitated microelectrodes were used to activate impedance transducers for the detection of H5N1 avian influenza virus. The aptamer–virus interaction caused measurable impedance changes. The signal was amplified with gold nanoparticles, showing a limit of detection of 0.25 HAU units for the purified virus samples and 1 HAU unit for the chicken tracheal swabs [62]. Single-stranded nucleic acid probes have been used as MREs to produce genosensors. Probe–target-sequence complementarity has been exploited for the specific binding and capture of targeted analytes. A reusable (5–7 times) genosensor capable of detecting *Escherichia coli* genomic DNA and cells within 14 min showed limits of detection (LOD) of 0.01 ng/μL and 11 cells/mL, respectively [63]. In another study, DNA probes were immobilized on palladium nanoparticles and then electrodeposited on a gold surface for the detection of *Brucella* DNA. The sensitivity of the sensor was 0.02 μA dm^3^/mol, the LOD was 2.7 × 10^−20^ mol dm^−3^, and the linear responses were recorded in a concentration range from 1.0 × 10^−12^ to 1.0 × 10^−19^ mol/dm^−3^ [64].

Field-effect transistors (FET) are capable of manipulating the flow of current in semiconductors and have high input impedance, characteristics which are exploitable in biosensing. For example, a potentiometric biosensor based on extended-gate FET was developed for the serological diagnosis of Bovine Herpes Virus-1 (BHV-1). The sensor utilized immobilized BHV-1 viral protein gE on its surface for the sensitive and selective detection of anti-gE antibodies in antiserum and serum samples. The sensor’s performance was similar to ELISA, enabling the detection of the analyte within 10 min. Additionally, the sensor was integrated into a chip with a microfluidic delivery system suitable for POC applications [65]. Similarly, a field-effect transistor (FET) biosensor was functionalized with α2,3- and α2,6-sialic acid-containing oligosaccharides (glycans) for the detection of H1 and H5 influenza A hemagglutinin, respectively. Used as MREs, the molecules, which were small in terms of Debye length, allowed the efficient detection of H1 and H5 on the FET sensor. The sensor was able to detect 60 H5 hemagglutinin molecules and 6000 H1 hemagglutinin molecules in 20-microliter samples [66].

In an effort to improve the performance of electrochemical biosensors, graphene and carbon nanotubes have been used to coat electrodes or nanoparticles, enhancing signal outputs [67,68]. Electro-reduced graphene oxide was used to produce electrochemically active dual-screen-printed electrode sensors for the detection of NEFA and beta hydroxyl-butyrate (βHBA) in dairy cows. Antibodies were used as MREs. Decreases in the electrochemical response of the sensor were attributed to the non-conducting behavior of the captured biomolecules. The sensor achieved a good correlation (R^2^ of 0.99) between signal responses and analyte concentration for both NEFA and βHBA in a concentration range from 0.1 mM to 10 mM [69]. In an alternative signal-enhancement technique, carbon nanotube biosensors were assembled on gold electrodes using layer-by-layer technology. The electrodes were previously patterned in the form of resistors onto a Si/SiO_2_ substrate. The sensors were functionalized with antibodies for the capture of avian metapneumovirus. Antibody-antigen interactions led to changes in the conductance of the sensor. The assay had LOD values of 10^2^ TCID_50_/mL [70].

Another signal enhancement approach is the use of nanoparticles. Polyethylene-glycol-coated and hyaluronic-acid-modified Fe_3_O/Au nanoparticles have been used to modify electrodes for the creation of an anti-fouling immunosensor. OMP31, a *Brucella* outer-membrane protein, was immobilized on the sensor’s surface for the detection of anti-Brucella antibodies in serum samples. The sensor responses and antibody concentration were linearly correlated in a concentration range from 10^–15^ g/mL to 10^–11^ g/mL. The LOD was 0.36 fg/mL [71]. In another study, a sandwich electrochemical immunoassay for the detection of *Salmonella pullorum* was developed. Antibody-functionalized silica-coated magnetic beads and secondary antibody-functionalized reduced graphene oxide coated with gold nanoparticles (electrochemical label) were used to capture the bacterial cells and form an immunocomplex. The immunocomplex was detected in the presence of 0.2 M HCl via differential pulse voltammetry, using a four-channel screen-printed carbon electrode. The assay had a linear response in a range from 10^2^ to 10^6^ CFU/mL and a LOD value of 89 CFU/mL, and it could be completed in less than 90 min [72].

Finally, magnetic nanoparticles coupled with biomolecules that facilitate the selective binding of the targeted analytes have been used for magnetic separation and, subsequently, the electrochemical detection of analytes [73]. Utilizing the principles of diagnostic magnetic resonance, a biosensor was developed for the detection of Gram (−) bacteria. Anti-LPS antibodies coupled with magnetic nanoparticles were used to capture Gram-negative (−) bacteria on a graphite ink electrode. The analytes were detected in real-time via conductometric measurements. The use of electromagnetic beads showed high sensitivity and selectivity in complex samples [74].

### 3.2. Optical Biosensors

**Figure 5:**
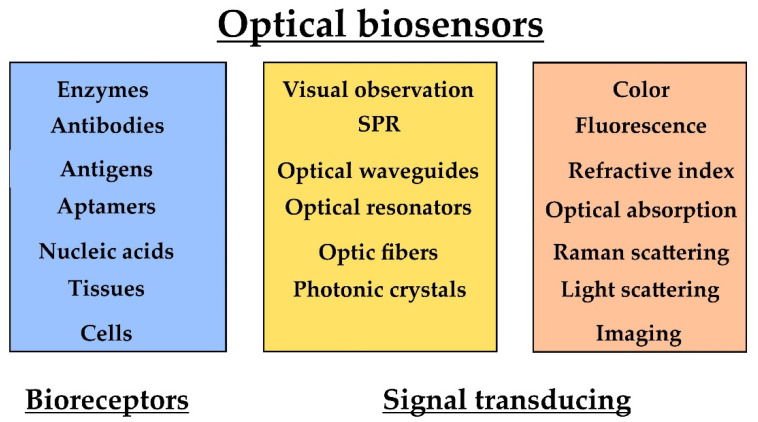
The basic components of optical biosensors. The interaction of the targeted analyte with the bioreceptors changes the optical properties of transducers.

These sensors rely on state-of-the-art optical biosensing technologies, such as surface plasmon resonance (SPR), optical waveguides and resonators, photonic crystals, and optic fibers [75]. Absorbance, reflectance, fluorescence, refractive index, and (chemi)luminescence measurements are utilized for the detection of biosensor–analyte interactions (Figure 5). Excellent reviews of optical biosensors have been published [75,76].

Numerous combinations of MREs and labels have been used for the biorecognition of analytes and signal generation in optical biosensing. Gold nanoparticles are among the most commonly used labels, especially in LFA applications [77]. In this context, aptamer-mediated isothermal strand-displacement amplification coupled with an LFA test was developed for the detection of *Salmonella enteritidis*. Initially, samples were incubated with two *S. enteritidis*-specific aptamers, an aptamer that mediated the isothermal amplification and a biotin-conjugated aptamer. The initial incubation was followed by a second incubation with streptavidin-modified magnetic nanoparticles and magnetic separation of the bacteria/aptamer complexes. The isothermal amplification products of the enriched samples (after the magnetic separation) were detected with an LFA biosensor that utilized Au-nanoparticle probes. The assay could detect 10 CFU/mL of *S. enteritidis*, allowing semi-quantitative detection with a strip reader [78]. In another study, peptide nucleic acid (PNA) and unmodified gold nanoparticles were used for the detection of avian Influenza viral RNA. Upon the introduction of complementary RNA to the PNA, PNA-induced gold nanoparticle agglomeration was prevented, resulting in a reduction in absorbance measured by a spectrophotometer. The assay could be completed within 15 min, had a visual LOD of 2.3 ng, and showed a specificity of 96.46% (95% CI = 93.8 to 98.2) and sensitivity of 82.41% (95% CI = 73.9 to 89.1) [79].

Fluorescent labels and dyes are another popular option for optical detection in biosensors. For example, DNAzymes (chemically active synthetic oligonucleotides) were conjugated with fluorescent dyes for the detection of bacteria. Upon interaction with bacterial lysates, DNAzymes were allosterically converted into active forms capable of cleaving fluorescent substrates and generating fluorescence signals. The reaction took place on paper-based sensors, which were used for the selective detection (within 5 min) of *Escherichia coli* in spiked milk, apple juice, and water samples, achieving LOD values of 100 cells/mL [80]. Following a different methodology, Sephadex renewable micro-columns were functionalized with the Fc fragment of purified human IgG antibodies for the capturing of *Staphylococcus aureus* bacteria via Fc/protein–A interactions. A secondary anti-protein A goat polyclonal antibody, conjugated with the Texas Red fluorescence marker, was used for the capturing of the previously formed complexes. The measurements were carried out with a FIALab 3500 B system, and the fluorescence was detected with an Ocean Optics USB 2000 instrument and a spectrophotometer. The LOD was 200 CFU/mL in the milk samples and the signal was linear in a range from 4 × 10^2^ to 4 × 10^7^ CFU/mL. The assay required 17 min to be completed [81].

The dielectrophoresis (DEP) force is generated by non-uniform electric fields and can manipulate dielectric particles based on their size. It has been combined with fluorescence for the detection of *E. coli* cells. The sensor utilized antibodies immobilized on the surface of gold tungsten microwires to capture the bacterial cells, after DEP-force manipulation. The antibodies captured the targeted bacterial cells and inhibited the binding of other materials on the sensor. Fluorescein-conjugated secondary antibodies were used in this case to quantify the captured bacteria [82].

Beyond classic fluorescent dyes, quantum dots (QDs), which are semiconducting nanoparticles of various chemical substances, have been recently used in biomedical applications [83]. QDs have been used for the detection β-hydroxybutyrate (βHBA), a key biomarker for the diagnosis of subclinical ketosis. The QDs were quenched via modification with nicotinamide adenine dinucleotide (NAD+). NAD+ enzymatically interacts with βHBA, forming NADH, which does not affect QD fluorescence. The reaction took place in a microfluidic chip integrated with a miniaturized, low-cost optical detection unit. The detection limits in serum and milk samples were 34.8 μM and 40.3 μM, respectively [84].

Standing at the crossroads of electrochemical and optical biosensing, carbon nanotubes were used to produce a near-infrared electrochemiluminesence sandwich immunosensor for the detection of porcine reproductive and respiratory syndrome virus (PRRSV). A glassy carbon electrode was modified in sequence with carbon nanotubes, CdTe/CdS quantum dots, chitosan, Au nanoparticles, and anti-PRRSV antibodies. Porous PtAu bimetallic nanotubes were used as near-infrared electrochemiluminesence catalysts. The PtAu nanotubes were modified with β-cyclodextrin and adamantine/anti-PRRSV antibody conjugates, capable of recognizing the previously captured viral antigens on the glassy carbon electrode and forming sandwich immunocomplexes. The LOD was 10.8 pg of viral antigens per mL [85].

Advances in surface plasmon resonance (SPR) have promoted optic-fiber sensing in the fields of life science, clinical diagnosis, medicine, and food safety [86]. Utilizing the principles of SPR, the substitution of single-strand DNA probes with locked nucleic-acid nucleotides (LNA) was used for the detection of the vp72 gene of African swine fever virus (ASFV). The assay was completed within 5 min, it allowed quantification in a range from 373 to 1058 copies/μL of genomic ASFV DNA, and the LOD and limit of quantification (LOQ) were 178 and 245 copies/μL of genomic viral DNA, respectively [87]. Using the same concept, a SPR biosensor assay for the detection of haptoglobin, a predictor of mastitis, was developed. Haptoglobin exhibits a strong interaction with hemoglobin. Milk samples were mixed with bovine hemoglobin and then applied on a haptoglobin-functionalized sensor. In the presence of haptoglobin in the milk samples, the hemoglobin molecules were bound and did not interact with the sensor (competitive format). The LOD of the assay was 1.1 mg/L [88].

Label-free optical biosensors are mainly based on refractive index measurements. This approach is compatible with transducing platforms, such as ring resonators, waveguides, surface plasmon resonance, fiber gratings, and photonic crystals [89]. Photonic integrated circuits (PICs) based on evanescent wave technology (ring resonators) were used for the detection of six swine viral pathogens. The PICs were functionalized with monoclonal and polyclonal antibodies. The antigen–antibody interactions resulted in measurable resonant shifts. The PICs were integrated in a microfluidic cartridge and were coupled with optic fibers and a laser for the detection of the analytes [90].

In another study, an excessively tilted fiber grating (Ex-TFG), inscribed in standard single-mode fiber, was used for the label-free detection of porcine circovirus type 2 (PCV-2). The sensor’s surface was functionalized with anti-PCV-2 monoclonal antibodies via staphylococcal Protein A. Upon laser excitation, changes in the refractive index were recorded with a fiber-optic grating demodulation system. The sensor showed a limit of detection (LOD) of ~9.371 TCID_50_/mL [91].

Finally, a biosensor based on high-spatial-resolution-imaging ellipsometry was integrated in a microfluidic reactor array system for the label-free detection of avian influenza virus H5N1. Silicon wafers were functionalized with Protein A and, subsequently, with the monoclonal antibody 4A4, which recognizes the H5N1 virus. After the bio-recognition event, the silicon wafers were analyzed with imaging ellipsometry. The LOD was 2.56 × 10^−3^ TCID_50_/mL and the assay could be completed in 10 min [92]. 

### 3.3. Piezoelectric Biosensors

Quartz crystal microbalance (QCM) sensors can be used to detect mass changes on the surfaces of quartz crystals. Piezoelectric disk-shaped crystals can be set into oscillation by electric current, producing shear waves that propagate perpendicularly to the crystal surface. The resonant frequency of the oscillation is proportional to the attached mass on the sensor’s surface [93]. Exploiting this phenomenon, a quartz crystal microbalance (QCM) immunosensor was used for the detection of influenza A and B viruses. The sensor was coupled with a flow injection system and an oscillator/frequency counter for signal detection. The sensor was functionalized with Protein A and, subsequently, with anti-influenza M1 antibodies. The LOD of the assay was 10^4^ PFU/mL. The conjugation of the detecting antibody with gold nanoparticles (diameter of 13 nm) reduced the LOD value to 10^3^ PFU/mL. The assay could be completed in less than an hour [94]. Similarly, a QCM sensor was modified with a nano-well structure (nano-porous gold film on a gold electrode) for the detection of H5N1 avian influenza virus, using aptamers as MREs. The detection range was from 2^−4^ to 24 hemagglutination units (HAUs)/50 μL [95]. Aptamers combined with QCM sensors have been also used for the detection of *Brucella melitensis* in milk. The aptamers were initially immobilized on magnetic particles (Fe_3_O_4_) for the magnetic separation and pre-concentration of the bacteria from liquid-sample solutions. The same aptamers were also used for the functionalization of the QCM chip. The assay had a detection limit of 100 CFU/mL, showing a linear response in a range from 10^2^ to 10^7^ CFU/mL. The magnetic particles could be recovered up to eight times [96].

### 3.4. Magnetic Biosensors

Magnetic nanoparticles have been used in biomedical applications, for both magnetic separation and signal transduction. For example, resonant coil magnetometers are capable of quantifying paramagnetic particles (PMPs). This approach was exploited for the detection of anti-PRRSV antibodies in serum in a competitive immunoassay format. Recombinant His-tagged, ORF 7 proteins were immobilized on a polysterene surface through mouse anti-His antibodies. PMPs were functionalized with anti-ORF 7 antibodies (SDOW17A), capable of recognizing the recombinant ORF 7 proteins. In the presence of sera containing anti-PRRSV antibodies, the coupling between the functionalized PMPs and the sensor’s surface was inhibited, resulting in a dose-dependent signal reduction. The assay was completed within 5 min and showed a sensitivity of 73% and a specificity of 100% [97].

In another work, giant magnetoresistance (GMR) sensors were functionalized with anti-NP influenza antibodies for the capturing of human- and swine-origin influenza A. Streptavidin-labeled magnetic nanoparticles were coupled with biotinylated monoclonal detection antibodies. In the presence of influenza viral particles, the magnetic nanoparticles came into proximity with the GMR sensors, altering their magnetization and, consequently, changing the resistance of the GMR sensors. The assay had an LOD of 1.5 × 10^2^ TCID_50_/mL and a saturation point of 1.0 × 10^5^ TCID_50_/mL [98].

Finally, wireless magnetoelastic (ME) biosensors have been modified with E2 phages as MREs for the detection of *Salmonella typhimurium* on eggshells. A ME resonator was used as the signal transducer. The system acts as a mass-sensitive biosensor that can be wirelessly actuated into mechanical resonance by an externally applied time-varying magnetic field. The mass increase on the sensor due to the phage–bacteria interaction resulted in a proportional decrease in the resonant frequency. The biosensors were incubated with the eggshells in a humidity-controlled chamber (95%) for 20 min. The detection limit was 1.6 × 10^2^ CFU/cm^2^ of eggshell surface [99].

### 3.5. Other Approaches in Signal Transduction

The cantilever biosensing principle is based on the mechanical stresses of cantilevers upon molecular binding. These stresses can cause the bending of sensors, described by Hooke’s law. Their deflection is directly proportional to the applied force and the cantilever spring constant. The spring constant determines the flexibility and sensitivity of the cantilever sensors. Reliable readouts are essential to exploit cantilever sensitivity; beam deflection or optical lever methods are commonly used. Cantilevers can be excited close to their resonance frequency and set into oscillation. When additional mass is attached on the oscillating cantilever, the resonance frequency is lowered, enabling the detection of the targeted analyte [100].

Sensors based on acoustic waves show good real-time monitoring capability and simplicity. The recognition event can be monitored through the changes occurring in the resonant frequency and motional resistance. Such an acoustic immunosensor has been developed for the detection of the herbicide, atrazine [101]. Another method to exploit acoustic waves in biosensing is acoustophoresis. Acoustophoresis on a microfluidic chip was used for label-free somatic cell cytometry in raw milk. The acoustophoresis removed lipid particles, thus eliminating the need for solvents, cell labeling, and centrifugation. Cytometry was performed in the lipid-free milk fractions either with a Coulter counter or with direct light microscopy [102].

Recently, the extended capabilities of smartphones, such as their user-friendliness, computational power, and easy data sharing, as well as their wide adoption, have made them particularly attractive for biosensing applications. Smartphones can be used both as optical (colorimetric, fluorescence, luminescence, surface plasmon resonance, spectroscopy, light scattering, and microscopy) and electrochemical biosensors. Their main drawback, uncontrolled or uneven light interference, has been addressed with the use of modules, such as collimating lenses and optical fibers. These modules minimize light dispersion and increase sensitivity during detection. Alternatively, computational methods or algorithms can be exploited to reduce both platform limitations and costs [103]. Table 1 summarizes the currently available electrochemical and optical biosensors for the detection of mastitis and important animal diseases.

## 4. POC Tests and Devices for Mastitis and Animal Diseases

Ideally, POC devices and tests should be user-friendly, portable, low-cost, efficient, capable of operating with small volumes of complex samples, and able to permit multiplexing [103]. Assays such as PCR, ELISA, LAMP, etc., have already been integrated into lab-on-chip devices and developments in biosensing have been exploited to produce novel POC devices and tests. Although promising, the proposed methods do not always coincide with the requirements of POC testing, as they may demand complex, off-chip handling (isolation of nucleic acids, labeling, etc.) or specialized reading equipment that is unavailable in animal production POC settings. In this review, we include POC devices that do not require centralized laboratories for the acquisition of valid results.

### 4.1. LFAs

The most commonly used readout labels in LFA tests are gold nanoparticles as they are easy to modify and allow simple visualization when they are accumulated on the test and control zones. For example, commercial LFA tests containing three test lines spotted with either anti-rhodamine antibodies, anti-fluorescein antibodies, or biotin-binding protein have been used for the multiplex detection of foot and mouth disease virus (FMDV) serotypes O, A, and Asia 1. Serotype-specific antibodies conjugated with either rhodamine, fluorescein, or streptavidin were used in this case to capture each serotype to its respective test line. Secondary, not serotype specific, anti-FMDV antibodies conjugated with colloidal gold were used for the labeling of each test line. The test could be completed within 15 min. The positive virus detection rates for serotypes O and A in lesion swabs of experimentally infected sheep were 38% and 50%, respectively [134]. Following a different approach, a pan-serotype strip test for the detection of FMDV was developed by exploiting a pan-serotype monoclonal antibody and recombinant bovine integrin αvβ6 (RBIαvβ6) as a universal capture ligand. In this case, the RBIαvβ6 was biotinylated and the anti-FMDV pan-serotype antibody was conjugated with colloidal gold. In the presence of FMDV, an RBIαvβ6/FMDV/antibody sandwich immunocomplex is formed and then captured at the test line by a biotin-binding antibody. Excess colloidal gold-antibody conjugates are captured at the control line by an anti-mouse antibody. This assay could be completed within 30 min. The strip was tested with 10% tissue suspensions and showed 100% specificity, a sensitivity similar to those of commercial ELISAs, and LOD values ranging from 3.7 to 5.4 log_10_ TCID_50_/0.1 mL, depending on the serotype tested [135].

Gold-nanoparticle-based LFA tests have been also used for serology. For example, a gold-nanoparticle-based LFA using recombinant proteins was developed for the serological diagnosis of the *Mycobacterium avium* subspecies, *paratubeculosis* in bovine sera. The recombinant protein MAP2963 (44 kDa) and Protein A are immobilized on the test and control lines, respectively. Gold nanoparticles are functionalized with guinea pig anti-bovine IgG antibodies that can form immunocomplexes with bovine anti-MAP2963 antibodies, which are then captured on the test line (MAP2963-spotted). Excess functionalized gold nanoparticles are captured in the control zone by Protein A. The assay was tested with 31 non-hemolyzed serum samples, and displayed LOD values of 1.98 μg/mL, a sensitivity of 84.2%, a specificity of 83.3%, and a positive predictive value (PPV) of 88.89%, and could be completed in less than 10 min [136].

Other bioreceptors have been used in LFAs, in addition to the typical conjugation of gold nanoparticles with antibodies for the detection of the targeted analytes. For example, two aptamers, J3APT and JH4APT, were capable of recognizing avian influenza H5N2 whole virus, forming sandwich complexes after the biorecognition event [137]. In this case, the J3APT aptamer was immobilized on the test line of the LFA test, whereas the JH4APT aptamer was labeled with gold nanoparticles and added onto the conjugate pad. The introduction of H5N2 viral particles led to the accumulation of labeled aptamer-virus complexes on the test line. This LFA showed LOD values of 6 × 10^5^ EID_50_/mL in buffer solutions and 1.2 × 10^6^ EID_50_/mL in duck fecal samples. An image analysis with ImageJ software reduced the LOD values to 1.27 × 10^5^ EID_50_/mL in the buffer and 2.09 × 10^5^ EID_50_/mL in the fecal samples [137]. On another LFA test, gold nanoparticles functionalized with monoclonal antibodies were coupled with QDs for the detection of avian influenza A. The antigen/monoclonal-antibody/gold-nanoparticle conjugates were dissolved from the test line of the LFA test using HCl–Br_2_ mixed solution and the generated gold ions were collected in a 96-well plate. The addition of CdTe QDs resulted in a reduction in the QD fluorescence due to the quenching ability of the gold ions. The fluorescence measurements were recorded with a microplate reader. This assay showed LOD values of 0.09 ng/mL, sensitivity of 100%, specificity of 88.2%, and efficiency of 90% when tested with clinical samples [138]. In another example, an LFA was developed using QDs functionalized with monoclonal antibodies for the detection of influenza A virus subtype H5 or H9. The QD-influenza conjugate was then captured at two distinct tests lines, forming sandwich immunocomplexes. Ultraviolet 365-nanometer excitation was used to record the fluorescence via a low-cost test-strip scanner, allowing sample quantification. The LFA-QD approach was tested with both serum and cloacal swab samples. The assay could be completed within 15 min and achieved LOD values of 0.016 HAU and 0.25 HAU for influenza A virus subtype H5 and H9, respectively [139].

Other promising labels for visual observation in LFAs are latex particles. For example, a commercial rapid immuno-chromatographic method, one of the few LFA tests for animal diseases to have undergone inter-laboratory evaluation, was developed for the detection of Bluetongue-virus-specific antibodies in animal sera. This test utilizes the Bluetongue virus VP7 protein immobilized on the test line. VP7-conjugated red latex microspheres were used for the formation of red immunocomplexes in the presence of anti-VP7-specific antibodies. Biotin-conjugated blue latex particles were captured by monoclonal antibodies on the control line. The assay showed a diagnostic sensitivity of 100% (95% C.I. (90.5–100)), specificity of 95.2% (95% C.I. (76.2–99.9)), repeatability (accordance), and reproducibility (concordance) for 100%-seropositive samples. The seronegative samples showed a repeatability of 45% and a reproducibility of 89%. The test achieved Cohen’s kappa values of 0.79 (95% CI (0.62–0.95)) in comparison with a commercial competitive ELISA [140].

Smartphones are useful tools in LFA diagnostics as they allow optical detection, imaging, and quantification. An LFA test combined with a smartphone-based optical detection method and latex beads was used for the detection of porcine pseudorabies virus (PRV) anti-gE antibodies. PRV-gE antibodies are useful to discriminate naturally infected from vaccinated animals, as commercial PRV vaccines are gE-deleted. The test line of the LFA was spotted with anti-gE antibodies and the control line with chicken IgY antibodies. Latex beads coated with PRV or goat anti-chicken IgY antibodies were used to form the test and control lines, respectively. In the presence of anti-gE antibodies, the immobilization of the latex beads/PRV conjugates on the test line was inhibited, resulting in a reduction in the optical transmittance on the test line. The optical transmittance measurements were performed using the smartphone’s ambient light sensor and a LED light incorporated in a 3D-printed reader. The images were analyzed using ImageJ. The assay could be completed within 15 min and showed a sensitivity of 100% and a specificity of 97.2%. When using real serum samples, the test displayed a 98% agreement with a commercial ELISA kit [141].

The simplicity and ease of operation of LFAs has led to their coupling with nucleic-acid amplification techniques to facilitate the simple visual observation of amplicons in the POC setting. For example, a LAMP assay was combined with an LFA test for the detection of ASFV DNA in blood and tissue samples from experimentally infected pigs. The LAMP amplicons were dual-labeled with biotin and fluorescein to enable their binding with latex beads and their capture on the test line of the LFA. The assay could be completed without the need for specialized equipment and achieved LOD values of 330 genome copies [142].

To simplify nucleic-acid extraction, fast technology analysis (FTA) cards and glass fiber were integrated into a lateral-flow strip for nucleic-acid extraction and the LAMP-based amplification of *E. coli* DNA. A portable, battery-powered heater was used for the LAMP isothermal amplification, enabling the use of the assay in resource-limited settings. Gold nanoparticles conjugated with an oligonucleotide detector probe were used for the visual detection of the LAMP products and smartphone-based quantification. The assay could be completed within 1 h and showed LOD values of 10–1000 CFU/mL in complex sample matrices [143].

In another study, recombinase polymerase amplification (RPA) was coupled with a streptavidin-coated gold nanoparticle-based LFA test for the detection of *Salmonella* enteritidis. DNA was extracted with boiling, after which the DNA extracts were centrifuged and amplified. The amplicons were conjugated with digoxin and streptavidin. The gold nanoparticles were coupled with the amplicons via streptavidin–biotin interactions and then immobilized on the test line via anti-digoxin antibodies. The control line contained anti-streptavidin antibodies to capture the excess gold nanoparticles. The LFA results were analyzed via imaging with a smartphone and the results were analyzed with a laptop for quantification. The assay showed LOD values of 91.4 CFU/mL and could be completed in less than 40 min without requiring laboratory equipment [144].

Capillary forces can be exploited to modify the standard setup of LFA tests. Following a vertical setup, a stacked-flow immunoassay was developed for the detection of *E. coli* bacteria. The samples were placed on a paper pad, facilitating migration on a conjugate pad containing HRP-labeled anti-*E. coli* antibodies. The formed complexes and excess HRP-labeled antibodies moved upward towards a blocking pad, which contained immobilized *E. coli* cells, thus blocking the excessive antibodies, allowing only the passage of the previously formed immunocomplexes on the conjugate pad. The immunocomplexes reached a substrate pad containing H_2_O_2_ and luminol and produced light enzymatically. The signal was captured with a CCD camera. The assay was completed within 5 min and achieved LOD values of 100 CFU/mL when tested with water samples [145]. In another publication, the method was modified with the use of TMB instead of luminol, achieving the same LOD values [146]. Table 2 summarizes the available LFA tests for the detection of animal pathogens.

### 4.2. Lab-on-Chip (LOC) Devices

LOC instruments are characterized by their ability to perform the whole analytical procedure in a single device and provide interpretable results. LOC devices are platforms that can be grouped based on the targeted analyte (whole cells, nucleic acids, or proteins). Cell-based LOC platforms are mainly used in animal production to estimate the somatic cell counts of milk, an indicator of mastitis and milk quality. For example, a rapid, low-cost, portable microfluidic differential-sedimentation cytometer was used to measure the somatic cell count and fat content of milk samples within 15 min. In this LOC device, 12 flattened funnel structures were fabricated on a rotating plastic compact disc. Each funnel structure could hold 150 μL of milk. Upon rotation, somatic cells were packed in a microfluidic channel, whereas fat globules accumulated towards the center of the disk, forming a “fat zone”. Two low-cost microscopes were used for the imaging of the cell pellet and the fat band. The device could accurately estimate cell counts in the range of 50,000–3,000,000 cells/mL [166].

In another study, an integrated, microfluidic cell counter designed to detect fluorescence was reported. The whole sample treatment was integrated in a microfluidic chip. A fluorescent cell dye was placed in a microfluidic chamber that manipulated milk samples through capillary action. Fluorescent-labeled somatic cells were quantified using a miniaturized, hand-held fluorescence measuring device. The fluorescence images were subsequently analyzed with custom software. The detection algorithm in this case showed an accuracy of 98.2% in a cell-count range of 100,000 to 300,000 cells/mL [167]. Using a similar concept, a fully integrated somatic-cell counter based on fluorescence detection was reported. In this case, samples are loaded in disposable plastic microchips. Ethidium bromide is used to stain the somatic cells. The fluorescence is recorded with a CCD camera coupled with a microscopic instrument. The captured images are analyzed with built-in software. The device yielded results that showed a high degree of correlation (R^2^ = 0.935 to 0.964) with results obtained from other commercial somatic cell counters when tested with composite milk samples [168].

Using a different approach, a LOC device utilizing microfluidics for the detection of *Streptococcus agalactiae* (Group B streptococci) and *Streptococcus uberis* in raw milk samples was reported. The method is based on super-paramagnetic nanoparticles functionalized with antibodies and magnetoresistive cytometry. The LOD of this device was 100 cfu/mL. The method showed a sensitivity of 73%, a specificity of 25%, and a PPV of 35% with the anti-*S. agalactiae* antibody, and a sensitivity of 41%, a specificity of 57%, and a PPV of 54% with the anti-GB streptococci antibody [169,170].

Nucleic-acid-based LOC platforms have received considerable attention in recent years, mainly due to their high sensitivity, which is attributed to nucleic-acid amplification. Indeed, the recent development of isothermal amplification has minimized the equipment requirements and seems promising for POC testing in low-resource settings. As an example, an assay designed for the surveillance of avian influenza in wild birds used a portable real-time RT-PCR (RAPID^®^ 7200) and dried reagents. This platform, apart from the portable PCR units, required a laptop for the analysis of the results and sample pretreatment for the isolation of nucleic acids (commercial kits). The assay, in this regard, could only be performed by trained personnel. In field conditions, the platform showed a 98% sensitivity and a 100% specificity [171].

In an alternative LOC platform, the whole process of a reverse-transcription LAMP for the detection of influenza A strains could be integrated on a polycarbonate disk. The disk contained microfluidic channels and chambers for centrifugal fluidic handling and the segregation of each analytic step. A heat plate and a miniaturized fluorescence detector were used for isothermal amplification and signal detection, respectively. The fluorescence was generated via calcein–magnesium-ion interaction. The device was capable of analyzing viral lysates and providing results within 47 min, showing the LOD values of 10 copies of viral RNA [172]. In another LAMP-based LOC device, a microfluidic chip was integrated with optic fibers and a fiber-optic sensor for the LAMP-based detection of pseudorabies virus (PRV). The microfluidic chip was incubated for 1 h in a water bath at 63 °C. DNA samples were extracted in the laboratory using a commercial kit. The LAMP products were detected in real time via absorbance or the optical observation of the turbidity. The assay required 0.4 μL of sample and showed LOD values of 10 fg [173]. Recombinase polymerase amplification (RPA) is particularly attractive for POC applications due to the lack of specific temperature requirements (performed at room temperature) for nucleic acid amplification. A RT-RPA assay for the detection of avian influenza H7N9 was incorporated into a portable laboratory named “Diagnostics-in-a-Suitcase”. Nucleic-acid isolation was performed with magnetic beads within 30 min. The RT-RPA was performed using a TwistAmp™ RT exo kit. The amplicons were detected with a fluorescence tube scanner (Twista). Isothermal reverse-transcription amplification was completed within 15 min and showed LOD values of 10 and 100 RNA copies per reaction for H7 and N9, respectively. The assay reagents could be stored at ambient temperature for up to 3 months and the device could be operated with a solar battery [174]. Following the same amplification technique, the DNA extraction, RPA, and detection of *Salmonella* were integrated in a disc-based centrifugal microfluidic device. A single laser was used for the wireless control of the valve actuation, cell lysis, and noncontact heating. The samples were enriched using antibody-functionalized magnetic beads and magnetic separation. The amplicons were detected with a simple LFA assay. The whole assay could be completed within 30 min, yielding LOD values of 10 CFU/mL and 100 CFU/mL in PBS and milk, respectively [175].

In another study, circular fluorescent probe-mediated, isothermal nucleic-acid amplification (CFPA) was integrated in a portable system for the detection of **ASFV**. CFPA is based on Bst DNA polymerase and structure-specific endonuclease 1 (FEN1) enzymes for the amplification and generation of a fluorescence signal. The sample distribution and detection were integrated in a single microfluidic chip. The DNA was extracted within 5 min using microbeads, a water bath, and a microcentrifuge. A portable hand-held fluorescence detection device was fabricated in-house for the quantification of the amplicons. All the necessary equipment could be encased in a lithium-powered suitcase. The results were uploaded to a cloud-based platform for the real-time monitoring of pigs. The detection system showed LOD values of 10 copies/μL, a 92.73% sensitivity, and a 100% specificity, and could be completed within 10–30 min [176].

Recently, cutting-edge molecular tools, such as the CRISPR-Cas12a, were integrated into microfluidic cartridges and used for the detection of ASFV DNA. The CRISPR-Cas12a was paired with a CRISPR RNA (crRNA). The ASFV DNA biorecognition resulted in the formation of a Cas12a/crRNA/ASFV DNA complex capable of cleaving a fluorescent single-stranded DNA (ssDNA) reporter. The fluorescence was recorded with a portable custom-designed fluorometer. The assay showed LOD values of 1 pM within 2 h. Due to its stability, the Cas12a/crRNA/ASFV DNA complex can remain active for 24 h, enabling detection up to 100 fM ASFV DNA. [177].

Protein-based LOC devices target the protein markers of diseases, such as enzymes and antigens. They lack amplification steps and usually require minimum sample pre-treatment. For example, a very simple volumetric microfluidic chip for the quantitative analysis of bovine catalase was developed. Catalase-spiked milk samples were mixed with H_2_O_2_ and immediately loaded in the chip. The produced O_2_ led to the propulsion of an ink bar, preloaded in a microfluidic channel. Smartphone images were analyzed to quantify the results. The chip showed LOD values of 20 μg/mL and the assay could be completed within 20 min. The chips could be fabricated within 3 min and their cost was $0.2 each [178]. Another LOC device for milk analysis, capable of monitoring milk pH, detecting *E. coli, Streptococcus agalactiae*, penicillin G, dihydrostreptomycin, and neutrophils was fabricated on PDMS. The device used SNARF-dextran as pH indicator and FITC-labeled antibodies to detect the other analytes via fluorescence microscopy. The assay was integrated in a microfluidic platform and could be completed within 2 h [179].

Sample contamination is common in farm conditions and often inhibits nucleic-acid amplification or produces false positive results. In an effort to minimize the effect of sample contamination and eliminate complex procedures, such as labeling, protein-based LOC devices utilizing advanced sensors based on refractive index measurements have been developed. For example, multiple high-precision planar Bragg gratings, serving as low-cost, robust refractive index sensors, were integrated in an optical microchip sensor for the rapid detection of foot and mouth disease virus (FMDV). The sensor surface was functionalized with monoclonal antibodies BF8, raised against FMDV type O1 Manisa, to capture the viral antigen in buffer solutions. The assay was integrated into a single portable device that provided both a simple readout (yes/no) and semi-quantitative information within minutes [180].

Surface plasmon resonance (SPR) is another sensitive and rapid method that has been incorporated into POC devices. A bioanalyzer, consisting of a micro-flow cell, a temperature regulator, an integrated biosensor (TSPR1k23), an optical platform, an electronic control unit incorporated into a photoelectric conversion device, and a universal serial bus (USB) interface circuit board, was developed for the SPR-based detection of infectious bursal disease virus. The antigen was captured on the sensor’s surface by monoclonal antibodies. The LOD of the bioanalyzer was 2.5 ng/mL in purified viral samples, diluted in PBS, and the assay could be completed within 20 min [181]. Following the same approach, anti-PCV2 antibodies were used to capture the Cap PCV-2 antigen in buffer solutions with a theoretical LOD of 0.04 µg/mL [182].

In another study, photonic integrated circuits (PICs), containing eight ring resonators each, were integrated into a microfluidic POC device for the detection of swine viral diseases. The ring resonators were functionalized with antibodies specific for each disease for the capturing of viral particles. Upon laser excitation, a shift in resonance was recorded in the monitored wavelength spectrum, caused by antibody–virus interaction on the sensor’s surface. The recorded data were uploaded on a cloud-based platform for storage and the real-time generation of results. The assay could be completed within 60 min. The device was capable of analyzing swine-oral-fluid field samples without requiring complex sample pretreatment. The sensors functionalized against porcine parvovirus (PPV) and porcine circovirus type 2 (PCV-2) showed sensitivity of 68.6% and 69.5%, specificity of 77.1% and 70.3%, and LOD values of 10^3^ viral copies/μL and 3.3 × 10^2^ copies/μL, respectively [183]. The sensors functionalized against PRRSV and swine influenza showed sensitivity of 83.5% and 81.8%, specificity of 77.8% and 82.2%, accuracy of 80.5% and 82%, precision of 77.6% and 84.9%, positive likelihood ratio (PLR) of 3.76 and 4.60, negative likelihood ratio (NLR) of 0.21 and 0.22, diagnostic odds ratio (DOR) values of 17.66 and 20.81, and LOD values of 3.3 × 10^2^ copies/μL and 3.3 × 10^1^ copies/μL, respectively [184].

## 5. Regulation of POC Tests for Animal Diseases

During the last 10 years, comprehensive reviews of novel biosensors and POC devices for veterinary use have been published. However, the regulatory and legislative aspects of POC testing are rarely discussed. An excellent work reviewing the current regulation was published on the OIE webpage by Potockova, Dohnal, and Thome-Kromer (2021). The level of regulatory monitoring differs across the globe, ranging from no regulation in the EU market to strict regulation, similar to that of human in vitro diagnostics, in Japan [185].

In the EU region, POC veterinary tests are only required to comply with general directives regarding products marketed within the union. More specifically, POC veterinary tests must comply with Directive 85/374/EEC, on product liability, and Directive 2001/95/EC, on product safety, whereas devices that use electricity (e.g., benchtop analyzers) must comply with Directives 2014/30/EU and 2014/35/EU on electromagnetic compatibility and low-voltage instruments, respectively [185]. Currently, there is no available legislation regarding the safety, quality, and performance of veterinary POC diagnostics. Markets, however, are regulated to some degree by EU individual states.

In the USA, veterinary POC devices and tests are defined in the Federal Food, Drug and Cosmetic Act, paragraph 321 h. POC devices do not require any specific clearance prior to marketing apart from validation; however, the FDA oversees the products and can act against false claims or misbranded products. Moreover, end users are encouraged to report adverse events related to the use of the tests. The jurisdiction over POC devices and tests for animal diseases is held by the Center of Veterinary Biologicals of the United States Department of Agriculture, according to the Virus-Serum-Toxin Act, Title 9, Code of Federal Regulation, Parts 101–104 [185,186]. The sensitivity, specificity, and reproducibility of POC tests must be validated across multiple laboratories to acquire licenses and reach the US market. POC tests for the official control and eradication of animal diseases, including large, well-defined animal populations, undergo secondary evaluation. Since 2018, license holders are obliged to maintain a registry of adverse events.

In Japan, POC tests are regulated by the Pharmaceutical and Medical Devices Act and are supervised by the Ministry of Agriculture, Forestry and Fisheries. The marketing of POC products requires the registration of the manufacturing plant to the local prefecture or the ministry for foreign manufacturers, the appointment of a marketing authorization holder, and approval for each marketed device. Compliance with the regulations is verified every 5 years by on-site visits from the competent authority’s delegates [185].

A unilateral approach to the regulations concerning the marketing and production of veterinary POC diagnostics may help to further assimilate cutting-edge technologies and diagnostic approaches in order to increase the impact of POC devices, especially in resource-limited settings. Moreover, the coordinated standardization of the performance requirements of POC tests may prevent poorly performing diagnostics from reaching the market and limit their negative impact on the management of animal diseases.

## 6. Challenges of Veterinary POC Testing

POC testing is becoming increasingly popular as an integral part of standard veterinary medical practice. This can be partly attributed to the popularization of POC testing in human medicine and, especially the COVID-19 crisis, which globalized LFA testing. Despite the success of POC tests in human medicine, its wide adoption in animal production faces many challenges due to the unique socioeconomic status of the sector.

Animal farming has a slim margin of profit, limiting the disposable income of farmers [8]. Consequently, investment in diagnostics or the prolonged use of POC devices by farmers are not the rule, with the exception of disease outbreaks. The development of a novel POC application requires substantial investment and funding for the research, validation, and marketing of the device. Given the limited market share, POC manufacturers usually lack the necessary financial incentives to commercially launch new POC devices or maintain a high supply of diagnostics in the market. In addition, intellectual property constraints and the unwillingness of private companies to share their technological advancements further hamper the transition of proof-of-concept prototypes to commercial devices [187]. Finally, the fabrication methods for the manufacture of POC devices are not always compatible with mass production, and the materials that POC devices usually comprise (glass, thermoplastics, etc.) are costly [188]. As a result, most POC products have prohibitive production costs for farm animal use [8].

Portability and field diagnosis are the essence of POC testing. However, many of the proposed POC methodologies fail to meet these requirements. Complex sample pretreatment (the isolation of nucleic acids, enrichment, labeling etc.) and handling, the integration level, the limited lifetime of reagents, device packaging and size, powering, user friendliness, the complex interpretation of the results, and data sharing are some of the most important factors that limit portability and POC testing implementation outside the limits of laboratories [189]. Processivity is another critical aspect of POC testing, as farmers or field veterinarians usually must test hundreds, or even thousands of animals for screening and epidemiological surveillance purposes [8]. Lastly, multiplexing is a desirable characteristic of POC devices and is often necessary to allow differential diagnoses, given that most animal diseases lack distinctive clinical signs and symptoms.

A multitude of the POC devices entering the market are not adequately validated [190]. Indeed, most validation studies fail to transparently provide essential information about the study design, the sample inclusion criteria, and the differences between the study and target populations, and they tend to present overly optimistic results [190]. Sensitivity and specificity, the two most commonly provided performance metrics, are intrinsic test characteristics and easily understood. However, they do not suffice to assess the post-test probability and interpret the test results. Moreover, they may be significantly different in the POC setting and/or outside the limits of well defined populations. To provide a more comprehensive view of the performance of a diagnostic test, other metrics, such as precision, accuracy, and the diagnostic odds ratio (prevalence-independent) should be provided [184,191]. The 95% confidence intervals of performance metrics are rarely presented, thus not allowing comparisons to other tests or robust evaluations of test performance. The samples used to evaluate diagnostics tests should cover the whole disease spectrum (including low analyte concentrations) to avoid the overestimation of performance metrics. For the same reason, clinical, complex sample matrices bearing common contaminants (particulate matter, blood, mucus, or feces) should also be included. Additionally, researchers should focus on improving on-chip, automated sample pretreatment and handling to successfully take POC devices to the field. Finally, disease epidemiology also plays a crucial role in the performance evaluation of POC devices. High prevalence in validation-study populations may artificially inflate predictive values, rendering these values unrealistic in low-prevalence settings [190]. For example, a test with 95% sensitivity and specificity will only produce a positive predictive value of 50% for a disease with 5% prevalence in the studied population.

On the other hand, farmers are often unable to exploit new avenues, and require extensive evidence before they decide to invest in new technologies [8]. Familiarizing farmers with sensor-based technologies in livestock management is imperative for the promotion of POC testing [192]. To successfully implement this goal, POC device manufacturers and traders should also provide the necessary framework, tailored to local conditions and disease epidemiology, for efficient POC testing. Since farmers usually lack the scientific background to interpret test results and fully exploit the added value of field diagnostics, they often rely on the expertise of veterinarians and animal scientists. For example, in a recent validation study of an LFA COVID-19 test in the UK, the recorded sensitivity was only 3.23% [193]. However, the test showed a negative predictive value of 99.17% due to the low prevalence (0.86%) in the studied population. The authors suggested that frequent, recurrent testing with the LFA test was necessary to detect COVID-19 cases [193]. Naturally, farmers cannot be expected to comprehend the complexity of this study and implement evidence-based POC testing and disease control strategies without proper guidelines from POC manufacturers and field veterinarians.

Finally, legislation, as previously stated, can play a pivotal role in the successful adoption of POC testing. Excessively strict legislation may prohibit the dissemination and development of novel technological solutions, as little financial incentive is provided to align with legal requirements, given the limited market share of animal diagnostics. On the other hand, the absence of regulation may lead to poor-performing devices reaching the market. This could damage the reputation of POC testing and have a negative impact on disease-control strategies, resulting in severe economic losses. A balanced approach would allow both the development and the marketing of novel POC devices, as well as protecting and safeguarding consumers.

## 7. Future Perspectives

With the integration of nanomaterials and novel instrumentation approaches, sensors and POC devices present exciting opportunities for the non-intrusive, real-time monitoring of animal health, behavior, and physiology [192]. Colloidal gold, noble metals, fluorescent and magnetic nanoparticles, quantum dots, nanozymes, conjugated polymers, surface-enhanced Raman scattering (SERS)-active nanomaterials, and carbon nanomaterials are only some of the nanomaterials that have been used for the labeling of LFAs to improve their sensitivity and facilitate their integration with novel miniaturized reading equipment [194,195,196]. In the same context, photothermal and photoacoustic methodologies exploiting the properties of plasmonic nanoparticles have been applied in LFA testing [197]. Molecularly imprinted polymers, carbon-allotrope-based nanomaterials, nanocages, nanoshells, and nanowires, nanostructured films and hydrogels, dendrimers, hyperbranched polymeric nanoparticles, and covalent organic frameworks are some of the novel materials that are used in biosensing, offering new opportunities for analyte detection [198,199].

Advanced materials and production techniques, such as the use of PDMS, thermoplastic elastomers and soft lithography, 3D printing, paper microfluidics, and the automated laser-printer deposition of hydrophobic ink, can be used for the mass production of POC devices [200,201]. Mass production can significantly reduce POC manufacturing costs and, consequently, testing costs. Moreover, mass production can help maintain a steady supply of POC diagnostics and cover the increasing demand. Besides maintaining production levels, the disposal of biological materials and waste is a further important aspect of POC testing. Microfluidic devices require small volumes of sample and reagents and reduce total waste. Waste is usually collected in a single tank, making disposal and disinfection easier. Paper-based devices can be easily and safely disposed by incineration [17]. These approaches can simplify waste control, reduce waste-management costs, and simultaneously minimize biohazards.

The miniaturization of reading equipment and POC test components in general is expected to lead to a higher level of integration and automation for POC devices [202]. Simple reading equipment from thermometers and pH meters, low-cost microscopes, SPR readers, and portable SERS readers have been used in POC devices to increase portability and enable sample analysis onto single platforms [202,203]. 3D-printed modules, mobile applications, and various accessories have been used to enhance the signal detection properties of smartphones, making them powerful platforms for POC testing. Smartphones have been used as instrumental interfaces, dongles, microscopes or test-result readers (brightfield, colorimetric, and fluorescent measurements) by exploiting their high-quality digital cameras, computer processors, touchscreen interfaces, wireless-data-transfer capabilities, and wide adoption [18,204]. Their data-transfer capabilities, along with cloud-based POC platforms, can enable data sharing to health centers and specialists for the acquisition of expert opinions and disease-management instructions, thus facilitating the development of telemedicine [205]. The integration of animal tracking and telemedicine can facilitate real-time epidemiological surveillance and evidence-based disease-control strategies.

Currently, simultaneous pathogen detection and subtyping usually requires centralized laboratories, specialized equipment, and trained personnel. As a result, veterinary health services usually lack multiplexed and easy-to-use field tests [187]. The detection of a single analyte may not be informative for the diagnosis of some diseases, and often does not suffice to assess the progress of diseases [206]. Multiplexed sensors and POC devices allow the detection of a multitude of discriminative biomarkers, thereby improving the accuracy of disease detection. Additionally, multiplexed tests in general reduce the required sample and reagent volumes and analysis times, require fewer materials, offer higher throughput, and facilitate the diagnosis of complex diseases [206,207]. Microarrays, antibody spotting, spatial multiplexing, time division, frequency division, and particle-based and barcoded multiplexing are some of the approaches used for the multiplexing of POC devices [206]. POC tests can be multiplexed relatively easily, without costly interventions, improving commercialization and leading to the de-centralization of disease diagnosis [187].

## 8. Conclusions

The emergence of novel pathogens, the modern farming systems, and the complexity of globalized supply chains and trade networks make animal production susceptible to disease outbreaks. Rapid, low-cost, and reliable field diagnosis is gradually becoming indispensable to support evidence-based disease-control strategies in veterinary medical practice. POC diagnostics will eventually achieve these goals by exploiting novel biosensing breakthroughs, advanced materials, and instrumentalization and mass production techniques. However, to do so, POC diagnostics must overcome a multitude of challenges. Firstly, veterinary POC diagnostics should focus on validation using complex clinical samples and large animal populations. All the necessary validation data and performance metrics, as well as their 95% CIs, should be available. Including samples from large animal populations that also represent the whole spectrum of the disease will help minimize the 95% CIs and reveal any methodological weaknesses. Secondly, POC diagnostics must be low-cost and simple. Only cost-effective POC diagnostics will be accepted by consumers and marketed successfully. Complex forms of handling, such as pipetting, sample pre-treatment, and nucleic-acid isolation, should be automated (on-chip) as end users lack the scientific background or time to perform these actions. Thirdly, POC devices should be portable and multiplexed. Farms usually lack the necessary infrastructure for permanently installed equipment, and different animal groups may be reared miles apart. On the other hand, farmers do not have the necessary financial means to perform a large number of tests or the necessary expertise for the differential diagnosis of animal diseases, making multiplexing imperative for successful and efficient POC testing. Indeed, most available POC devices target only few and relatively simple analytes, such as enzymes, proteins, and viruses, to achieve optimal performances. Only a few POC devices targeting bacteria, protozoa, and parasites have been developed, despite the fact that these pathogens are much more likely to break the species barrier and infect humans. Future steps should focus on the creation of interdisciplinary research teams, including animal experts, that are able to fully exploit and integrate the recent technological advancements into animal production. Additionally, public funding and the collaboration of the public and private sectors are required to support the research and development of novel POC diagnostics. Moreover, private companies should be given the necessary financial incentives to undertake the commercialization of novel POC devices and tests. Finally, a unilateral and flexible legal framework would facilitate the commercialization of POC devices and the dissemination of novel technologies, as well as safeguarding consumers. Overcoming these issues and challenges could finally create the era of POC testing, telemedicine, and precision farming that has long been envisioned.

## Figures and Tables

**Table 1:** Available electrochemical and optical biosensors for the detection of mastitis and animal diseases. The available literature for the remaining transducing options is included in the main text.

Targeted Analyte	Recognition Element	Materials	Detection Technique	Detection Matrix	Performance	Reference
		***Electrochemical biosensors***				
Haptoglobin	Goat anti-bovine Hp polyclonal antibody (Abcam)	Functionalized gold electrode	Amperometric detection	Skimmed milk	LOD ^1^ of 0.63 ng/mL. Linear response range: 15–100 mg/L. Detection in 5 min	[104]
Haptoglobin	Anti-Hp antibody	Functionalized liquid-exfoliated two-dimensional phosphorene (Ph) nanosheets electrodeposited on screen-printed electrode	Differential pulse voltammetry	Spiked serum samples	LOD of 11 ng/mL. Linear response range: 10–10 × 10^3^ ng/mL. Detection in 60 s	[105]
	Mouse polyclonal anti-M1 antibodies	Functionalized gold electrodes	Electrochemical impedance spectroscopy	PBS ^2^ buffer	LOD of 2 × 10^−2^ ng/mL. Detection in 30 min	[106]
Avian influenza A H5N1 virus	Monoclonal antibodies (produced in mouse myeloma cells)	Protein-A-modified interdigitated array microelectrode	Electrochemical impedance spectroscopy	Tracheal and cloacal swabs	LOD of 2^−1^ HAU ^3^/50 μL. Linear response range: 2^−1^–2^4^ HAU/50 μL. Detection in 1 h	[107]
Avian influenza A H5N1 virus	H5N1-specific aptamer	Aptamer-modified magnetic beads, concanavalin A-glucose oxidase-Au nanoparticle complexes, glucose solution, screen-printed interdigitated array electrode	Electrochemical impedance spectroscopy	PBS buffer	LOD of 8 × 10^–4^ HAU in 200 μL	[108]
Avian influenza A H7N1 virus	Rabbit anti-H7N1 polyclonal antibodies (affinity-chromatography purified)	Functionalized gold electrodes	Electrochemical impedance spectroscopy	Antigen extracted from vaccine diluted in buffer	LOD of 5 × 10^3^ ng/mL	[109]
Avian influenza A H7N9 single-stranded (ss)DNA	DNA tetrahedral probe	Biotinylated-ssDNA oligonucleotide (detection probe), avidin–horseradish peroxidase (HRP)	Amperometric detection	ssDNA (PCR product in buffer)	Sensitivity of 10^−7^ μM. Detection in under 80 min	[110]
Quantum-dot-modified influenza hemagglutinin	Biotinylated glycans	Streptavidin-modified magnetic particles, glassy carbon microelectrode, 3D microfluidic chip	Differential pulse voltammetry	Vaccine hemagglutinin in buffer	Accuracy 80%. Linear response range: 60–500 μM. Detection in 45 min	[111]
Bovine viral diarrhea (BVD) antibodies	BVD virus	Functionalized nanowire sensor integrated on chip	Electrochemical impedance spectroscopy, cyclic voltammetry	Serum	Detection of 10^3^ ng/mL. Detection in 20 min	[112]
BVD virus, anti-BVD antibodies	BVDV-1 monoclonal antibody (RAE0823), recombinant purified BVDV-1 Erns protein (BVDR16-R-10)	Six gold nanoband electrodes, silicon-chip-based biosensor platform	Electrochemical impedance spectroscopy	Serum	Detection in 20 min	[113]
Fowl adenovirus-9	Anti-adenovirus, group II polyclonal antibody	Functionalized graphene quantum dots, functionalized gold nanobundles, carbon electrodes, UV–visible light irradiation	Voltammetry, local electric signal enhancement by light–matter interaction (graphene-mediated)	Serum	LOD of 10 PFU ^4^/mL in buffer and 50 PFU/mL	[114]
Protective antigen (Anthrax biomarker)	Short-chain peptide	Functionalized gold electrodes	Square-wave voltammetry	Antigen diluted in PBS + BSA ^5^	LOD of 5.2 × 10^−6^ μM. Detection in 60–100 min	[115]
*Streptococcus suis* serotype 2	Antibodies (sandwich immunoassay)	Antibodies immobilized on gold nanoparticles electrodeposited on a glassy carbon electrode, l-cysteine/hollow PtPd nanochains/glucose oxidase/antibody bioconjugates (HRP-mimicking), d-glucose solution	Peroxydisulfate electrochemiluminescence	Antigen diluted in serum	LOD of 33 × 10^−6^ ng/mL. Linear response range: 0.0001–100 ng/mL. Detection in 40 min	[116]
Gram-negative bacteria	Anti-LPS antibodies (mouse monoclonal and goat polyclonal, Abcam)	Functionalized magnetic nanoparticles, interdigitated microelectrodes	Conductometry	1% serum in PBS	Detection range: 10–10^3^ CFU ^6^/mL	[117]
*Salmonella* spp.	Anti-Salmonella magnetic beads (prod. no. 710.02, Dynal Biotech). Anti-Salmonella-HRP (rabbit polyclonal, prod. no. ab20771, Abcam)	Antibody-functionalized magnetic particle, polyclonal anti-Salmonella-HRP antibody, graphite-epoxy composite magneto-sensor	Amperometric detection	Skimmed milk	LOD of 7.5 × 10^3^ CFU/mL. Detection in 50 min	[118]
*Brucella melitensis*	Anti-brucella antibodies	Gold nanoparticle-modified screen-printed carbon electrodes	Cyclic voltammetry, electrochemical impedance spectroscopy	Milk	LOD of 4 × 10^5^ CFU/mL. Linear response range: 4 × 10^4^–4 × 10^6^ CFU/mL. Detection in 90 min	[119]
*Brucella abortus*	Anti-lipopolysaccharide antibody (Abcam 3535)	Screen-printed gold-plated electrodes, copper-doped nickel and zirconium oxide nanoparticles.	Cyclic voltammetry, electrochemical impedance spectroscopy	Phosphate buffer	Detection range: 10^3^ CFU/mL–2 × 10^6^ CFU/mL	[120]
*Babesia bovis* circulating antibodies	Recombinant version of the C-terminal portion of RAP-1 (Portuguese *B. bovis* Santarém strain)	Functionalized gold electrodes	Electrochemical impedance spectroscopy	Serum	Detection range: 16.7–500 μM	[121]
		***Optical biosensors***				
Influenza A H1N1 virus	FAM-labeled aptamers	Aptamer-modified magnetic beads for magnetic separation, fully integrated microfluidic chip, optical detection unit	Fluorescent measurements	PBS	LOD of 0.032 HAU units. Detection in 30 min	[122]
Swine-origin influenza A H1N1 virus	Anti-H1 antibody (ProSci, Poway, CA, USA)	SPR chip (BK7 glass slide coated with a laminated Ag/Au 37/8 nm, metal layer), paired-surface plasma-wave biosensor	Surface plasmon resonance (SPR)	Mimic solution (human mucosa in PBS)	Theoretical LOD of 30 PFU/mL, 1.8 × 10^2^ PFU/mL. Detection in 20 min	[123]
Avian influenza A H5N1 virus	Anti-H5N1 hemagglutinin antibody 2B7 (ab135382), anti-H5N1 neuraminidase polyclonal antibody (Cat. PA5-34949)	Anti-H5N1 hemagglutinin antibody functionalized chiral gold nanohybrids, anti-H5N1 neuraminidase functionalized quantum dots	Circular dichroism spectra	Serum	LOD of 10^−3^ ng/mL	[124]
Infectious bronchitis virus (IBV)	Anti-IBV antibodies	Alexa Fluor 488 labeled anti-IBV antibody, anti-IBV antibody conjugated with molybdenum disulfide (quencher) and immobilized on a cotton-thread-based microfluidic platform	Fluorescence-resonance energy transfer (FRET)	Serum	LOD of 4.6 × 10^2^ EID_50_ ^7^/mL. Linear response range: 10^2^–10^6^ EID_50_/mL	[125]
Muscovy duck parvovirus	ssDNA aptamer	Unmodified gold nanoparticles	Spectrophotometry or visual observation	Allantoic fluids	LOD of 1.5 EID_50_ for spectrophotometry or 3 EID_50_ for visual observation. Detection in 70 min	[126]
PRRSV ^9^	Anti-PRRSV monoclonal antibody (SDOW17)	Fluorescent (Alexa Fluor 546) labeled antibody/Protein A/gold nanoparticles or quantum dots (catskill green) complexes	Fluorescence resonance energy transfer (FRET)	PBS	Detection limit of 3 viral particles/μl	[127]
PRRSV	Anti-PRRSV antibody (lgG2b isotype)	CdTe:Zn2^+^ quantum dots, antibody modified platinum nanotubes (quencher)	Fluorescence	Serum diluted in PBS	LOD of 2.4 ng/mL. Linear response range: 5.6 ng/mL–66.6 ng/mL	[128]
Bovine viral diarrhea (BVD) virus	Anti-BVD virus monoclonal antibodies (9021 Jeno Biotech or 244-FA National Veterinary Service Laboratories, USA	Functionalized highly carboxylated polystyrene microparticles, y-channel microfluidic chip with optical fibers	Static forward light scattering	Tissue culture media and fetal calf serum diluted in PBS	LOD of 10 TCID_50_ ^8^/mL	[129]
Foot and mouth disease (FMD) antibodies	FMD antigen (O, A and Asia-1 serotypes from commercial vaccine)	Anti-bovine IgG functionalized gold nanoparticles, nitrocellulose or nylon membrane	Dot-blot assay, visual observation	Serum	10^−4^ dilution of serum samples	[130]
Vesicular stomatitis virus (VSV)	Anti-VSV-G (monoclonal 8G5, monoclonal 1E9), anti-VSV-M (monoclonal 23H12), anti-VSV-N (monoclonal 10G4)	Interferometric reflectance imaging sensor (IRIS), thermally grown SiO_2_ on Si, CCD camera	Spectral reflectance imaging	Cell lysate	3.5 × 10^5^ PFU/mL	[131]
*Brucella* DNA	Nucleotide probe	Ionic self-assembled multilayer, long-period grating optical fiber	Optical spectrum analysis of the refractive index	Culture and tissue lysates	LOD of 100 cells/mL. Detection in 30 min	[132]
*Salmonella typhimurium*	Goat anti-Salmonella antibodies (Kirkegaard and Perry Laboratories)	Labeled (donor Alexa Fluor 546) anti-Salmonella antibodies, labeled (acceptor Alexa Fluor 594) protein G, fiber-optic biosensor	Fluorescence resonance energy transfer (FRET)	Ground pork	LOD of 10^5^ CFU/g of ground pork. Detection in 5 min	[133]

^1^ LOD: limit of detection, ^2^ PBS: phosphate buffered saline, ^3^ HAU: hemagglutination units, ^4^ PFU: plaque-forming units, ^5^ BSA: bovine serum albumin, ^6^ CFU: colony-forming units, ^7^ EID_50_: 50% egg infection dose, ^8^ TCID_50_: median tissue culture infectious dose, ^9^ PRRSV: porcine reproductive and respiratory syndrome virus.

**Table 2:** Available LFA tests for the detection of animal pathogens.

Targeted Analyte	Materials and Methods	Equipment	Samples and Handling	Performance	Reference
PRV	Fluorescent immunochromatographic strip, anti-PRV gB monoclonal antibodies, 3D-printed customized pocket fluorescence observation instrument	None	Homogenized pig tissues	LOD of 0.13 ng/mL. Detection within 13 min	[147]
Porcine epidemic diarrhea virus (PEDV)	LFA test, antibody-functionalized gold nanoparticles, 3D-printed transmittance reader, image analysis	Smartphone	PEDV solution	LOD of 55 ng/mL. Linear detection range: 78–20 × 10^3^ ng/mL	[148]
Bovine ephemeral fever virus (BEFV)	RPA ^1^, FAM ^2^, and biotin labeled amplicons, LFA	TwistAmp NFO kit for RPA amplification, heat block	RNA isolation from clinical samples and reverse transcription	LOD of eight copies per reaction. Coincidence rate with real-time PCR of 96.09%. Detection in 25 min	[149]
BVDV	Immunochromato-graphic test strip, anti-NS3 monoclonal antibody 46/1-conjugated gold nanoparticles	None	Leukocyte extracts	Sensitivity and specificity of 100% and 97.2%, respectively. Detection in 15 min	[150]
FMDV	LFA test, gold nanoparticles, monoclonal anti-FMDV antibody 1F10 or 2H6	None	Homogenized epithelial suspensions	Sensitivity of 84% for 1F10 and 88% for 2H6. Specificity of 99% for both antibodies	[151,152]
FMDV viral RNA	RT-LAMP, FIP ^3^ and BIP ^4^ labeling at the 5′ terminus with fluorescein and biotin, LFA test	Water bath	RNA, epithelial suspensions spiked with FMD virus, epithelial samples, air samples, RNA isolation	LOD of 10 viral copies	[153]
FMDV viral RNA	RT-RPA-, FAM-, and biotin-labeled amplicons, LFA	TwistAmp NFO kit for RPA amplification, water bath	cDNA, reverse transcription, RNA isolation	LOD of 10 copies (plasmid DNA), 98.6% concordance with real-time PCR	[154]
ASFV DNA	RPA, FITC ^5^, and biotin labeled amplicons, LFA	TwistAmp NFO kit for RPA amplification, thermocycler	DNA isolated with a magnetic bead-based kit	Positive agreement of 100% with PCR. Detection in 15 min	[155]
Classical Swine Fever (CSFV)	Fluorescent microsphere (FM)-based LFA, monoclonal-antibody-functionalized FMs	Fluorescent immunochromatographic strip reader, fluorescent camera	Tissue extracts	LOD of 5.28 ng/mL, positive coincidence rate, negative coincidence rate, and total coincidence rate of 95.8%, 100%, and 98%, respectively. Detection within 15 min	[156]
CSFV RNA	RT-LAMP-, DIG ^6^-, and FITC-labeled amplicons, LFA	Thermocycler	Cell-culture supernatants, serum, RNA isolation	LOD of 100 copies per reaction. Detection in 70 min	[157]
PCV-2 antibodies	Immunochromatographic test strip, recombinant-Cap-protein-labeled colloidal gold	None	Serum samples	Agreement of 94% with commercial ELISA. Sensitivity and specificity of 93.14% and 98.70%, respectively. Detection in 5 min	[158]
PRRSV antibodies	Immunochromatographic test strip, PRRSV recombinant membrane and nucleocapsid proteins, Protein-G-conjugated gold nanoparticles	None	Serum samples	Sensitivity of 98.6%, specificity of 97.8%, accuracy of 98.3%	[159]
*Salmonella hilA* gene	LAMP-, FITC-, and biotin-labeled amplicons, LFA test	Heating block	DNA isolated with commercial kit	LOD values of 13.5 × 10^−3^ ng/mL of genomic DNA and 6.7 CFU/mL. Detection in 40 min	[160]
*Salmonella* Typhimurium DNA	RPA-, DIG-, and FAM-labeled amplicons, LFA	TwistAmp RPA reaction kit. Thermostatic water bath	DNA isolated with commercial kit	LOD of 10^−6^ ng (genomic DNA) and 1.95 CFU/mL in milk samples. Detection in less than 20 min	[161]
*Brucella* spp.	Multiple cross-displacement amplification, FITC- and biotin-labeling of amplicons, LFA utilizing dye streptavidin-coated polymer nanoparticles	Water bath or heat block	Human- and goat-serum samples, DNA extraction	LOD of 10^−5^ ng of templates (pure cultures). Detection in 70 min	[162]
*Campylobacter jejuni* and *Campylobacter coli*	LFA test, gold nanoparticles, monoclonal mouse anti–Campylobacter A and/or B	None	Chicken feces, dilution with saline, filtration, sedimentation for 10 min	LOD of 6.7 log CFU/g for *Campylobacter jejuni* or 7.1 log CFU/g for *Campylobacter coli*. Detection in 20 min	[163]
*Mycobacterium avium* subsp. *paratuberculosis*	RPA, labeled amplicons, LFA	TwistAmp RPA reaction kit, thermostatic water tank	DNA extracted with commercial kit	LOD of eight copies per reaction. Sensitivity and specificity of 100% and 97.63%, respectively. Detection in 35 min	[164]
*Mycoplasma ovipneumoniae* DNA	LAMP-, DIG-, and biotin-labeled amplicons, LFA	Water bath	Lung tissue sample, DNA extraction	LOD of 100 CFU/mL. Sensitivity of 86% in clinical samples, Detection in 70 min	[165]

^1^ RPA: recombinase polymerase amplification, ^2^ FAM: fluorescein amidite, ^3^ FIP: forward inner primer, ^4^ BIP: backward inner primer, ^5^ FITC: fluorescein isothiocyanate, ^6^ DIG: digoxigenin.

## Data Availability

Not applicable.
